# Luminescent Assay for the Screening of SARS‐CoV‐2 M^Pro^ Inhibitors

**DOI:** 10.1002/cbic.202200190

**Published:** 2022-06-14

**Authors:** Daan Sondag, Jona Merx, Emiel Rossing, Thomas J. Boltje, Dennis W. P. M. Löwik, Frank H. T. Nelissen, Mark van Geffen, Cornelis van 't Veer, Waander L. van Heerde, Floris P. J. T. Rutjes

**Affiliations:** ^1^ Institute for Molecules and Materials Radboud University 6525 AJ Nijmegen The Netherlands; ^2^ Enzyre BV, Novio Tech Campus Transistorweg 5-i 6534 AT Nijmegen The Netherlands; ^3^ Department of Haematology Radboud University Medical Centre Nijmegen The Netherlands) and Haemophilia Treatment Centre Nijmegen Eindhoven Maastricht (HTC-NEM) (The Netherlands

**Keywords:** enzymes, inhibitors, imaging agents, luminescence

## Abstract

Since the outbreak of SARS‐CoV‐2 in December 2019 millions of infections have been reported globally. The viral chymotrypsin‐like main protease (M^Pro^) exhibits a crucial role in viral replication and represents a relevant target for antiviral drug development. In order to screen potential M^Pro^ inhibitors we developed a luminescent assay using a peptide based probe containing a cleavage site specific for M^Pro^. This assay was validated showing IC_50_ values similar to those reported in the literature for known M^Pro^ inhibitors and can be used to screen new inhibitors.

## Graphical abstract

**A luminescent assay** for the main protease M^Pro^ of SARS‐CoV‐2 was developed and subsequently used for the screening of inhibitors of the chymotrypsin‐like protease. The caged luminescent peptide probe used in this study releases photons upon proteolytic activity of M^Pro^ in the presence of luciferase.

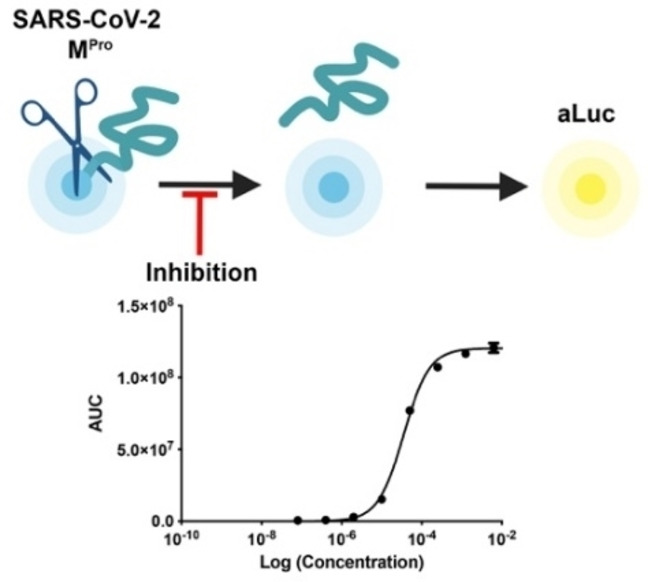

## Introduction

In December 2019 an outbreak of pneumonia with flu‐like symptoms rapidly spread across Wuhan, China.^[1]^ The severe acute respiratory syndrome corona virus 2 (SARS‐CoV‐2) led to a major global outbreak of the contagious COVID‐19. The novel beta‐coronavirus is suspected to originate from bats considering its genome is over 96 % identical to bat CoV RaTG13, whereas it is only 79 % similar to SARS‐CoV‐1.^[2]^ The outbreak caused a global pandemic of the infectious disease and had a tremendous impact leading to over 400 million known cases, and this is still rapidly increasing.^[3]^ Fortunately, prophylactic vaccines are now available which can prevent COVID‐19 infections or decrease disease severity.^[4]^ However, the efficacy of COVID‐19 vaccines may be compromised by the appearance of new virus variants and hence the development of broad spectrum antiviral compounds to treat SARS‐CoV‐2 infections remains of high societal relevance.^[5]^ Along with the structural spike protein (S), nucleocapsid protein (N), membrane (M) and envelope protein (E) of SARS‐CoV‐2, the two viral chymotrypsin‐like M^Pro^ (main protease; 3CL^Pro^, nsp5) and PL^Pro^ (papain like protease; nsp3) proteases perform a crucial role in viral replication. Cellular entry of the virus depends on binding of the viral spike (S) protein to the ACE2 receptor expressed in the host.^[6]^ Subsequent S protein priming is initiated by the host cell transmembrane protease serine 2 (*TMPRSS2*).^[7]^ After viral entry, M^Pro^ and PL^Pro^ are both involved in viral gene expression and replication through a complex cascade involving the proteolytic processing of replicase polyproteins (pp).^[8]^ The cysteine protease M^Pro^ acts on at least 11 conserved sites on the large replicase polyproteins 1ab and pp1b, predominantly on ‐LQ↓SAG‐ sequences (↓ indicates cleavage site).^[9]^ The generated polyproteins are crucial for the synthesis of the viral RNA and proteins.^[10]^ PL^Pro^ enables the viral spread of the virus *via* generation of functional complexes and cleaves pp1a and pp1b recognizing ‐LXGG↓ sequences.^[11]^ Besides the proteolytic activity of PL^Pro^ on the polyproteins, it exhibits far more dominant functions such as deubiquitinating activity and the cleavage of interferon‐stimulated gene 15 (*ISG15*).^[12]^ The crucial and distinct role of M^Pro^ in viral replication and infection along with the lack of closely related human homologues, makes it an attractive target for the development of antiviral drugs.^[13]^ To this end, biochemical M^Pro^ assays are needed to identify inhibitors of this viral enzyme. Most established assays employ internally quenched fluorescent substrates.^[14]^ Recent efforts by Rut and co‐workers identified the substrate profile of both M^Pro^ and PL^Pro^ using a combinatorial library of fluorogenic substrates and subsequent patient‐sample imaging employing immunofluorescence.^[15]^ The amino acid sequence preference observed for both M^Pro^ and PL^Pro^ served as a basis for the design of potential viral inhibitors or for the development of diagnostic probes.

**Figure 1:**
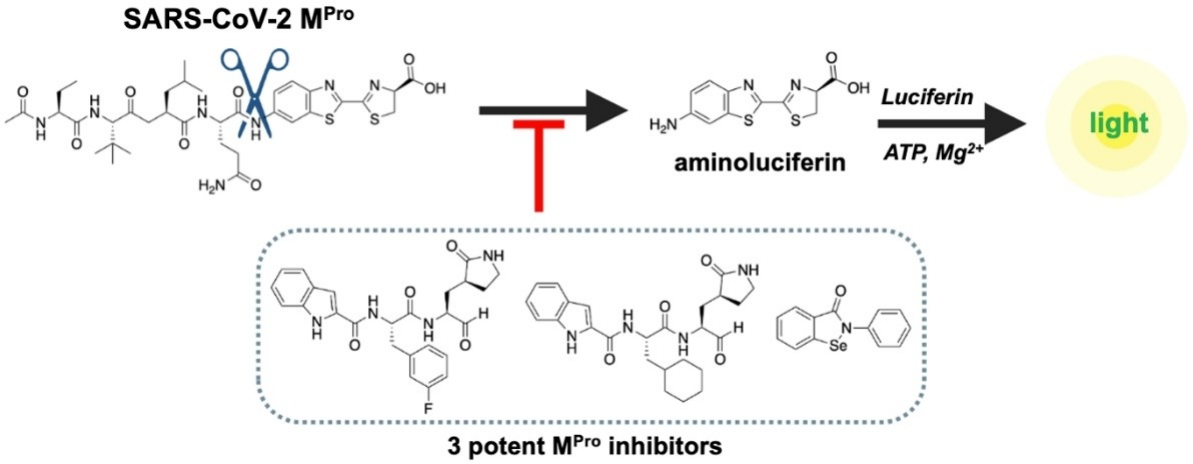
Caged aminoluciferin strategy involving proteolytic scission of the peptide substrate causing release of aminoluciferin. This is subsequently converted into a light signal proportional to the activity of M^Pro^ by luciferase. Inhibition of M^Pro^ leads to a corresponding decrease in the generated light signal, which can be quantified.

Luminescence imaging has emerged as a powerful technique to monitor enzymatic activities and abrogates the need for an external light source.^[16]^ Consequently, luminescent imaging does not suffer from autofluorescence nor photobleaching.^[17]^ Herein we report an enzymatic assay platform for the rapid detection of SARS‐CoV‐2 M^Pro^ activity using a chemiluminescent approach affording a real‐time readout. A peptide caged aminoluciferin (aLuc) substrate specific for M^Pro^ was synthesized. Upon proteolytic cleavage of the latent peptide substrate by M^Pro^, aLuc is converted into a proportional light signal by the action of firefly luciferase (Figure 1). This assay proved to be highly sensitive and generated a measurable and quantifiable signal at low nanomolar concentrations resulting from M^Pro^ activity. Screening of a number of known M^Pro^ inhibitors accurately reproduced IC_50_ values reported in the literature demonstrating that our assay is robust. Finally, the use of luminescence instead of fluorescence renders our approach suitable for potential miniaturization as no external light source is needed and the generated light signal can directly be detected by a small photon sensor.

## Results and Discussion

**Scheme 1:**
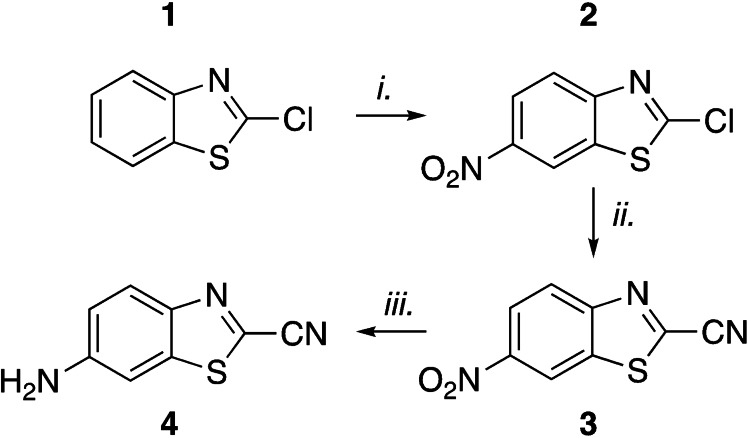
Synthesis of 6‐aminobenzo[*d*]thiazole‐2‐carbonitrile (**4**). Reagents and conditions: i. KNO_3_ (1.1 equiv), H_2_SO_4_, 89 %. ii. NaCN (1.1 equiv), DABCO (0.15 equiv), ACN, 81 %. iii. Fe (50 equiv), AcOH, 53 %.

In the fluorescent profiling study by Rut and co‐workers Ac‐Abu‐Tle‐Leu‐Gln‐7‐amino‐4‐carbamoylmethylcoumarin was found as the preferred substrate for M^Pro^.^18^ Based on this finding we designed the activity based luminescent probe **8** containing this M^Pro^ specific proteolytic site. The specificity of M^Pro^ is highly conserved among the SARS‐CoV‐1 and SARS‐CoV‐2 viruses and also seen back in the numerous of crystal structures available.^[18] [19]^ Synthesis of the aLuc precursor was achieved by the reaction sequence shown in Scheme 1 based on the described scalable method by Bon and co‐workers.^[20]^ Commercially available 2‐chlorobenzothiazole (**1**) was nitrated with potassium nitrate in sulfuric acid to give nitrobenzothiazole **2**, which was readily purified by recrystallization. The nitrile moiety was introduced with sodium cyanide catalyzed by 1,4‐diazabicyclo[2.2.2]octane (DABCO) to afford nitrile **3**. Finally, the nitro group was reduced to the corresponding aniline with iron and acetic acid to afford 6‐aminobenzo[*d*]thiazole‐2‐carbonitrile (6‐ABTC, **4**), which is the aLuc precursor.

**Scheme 2:**
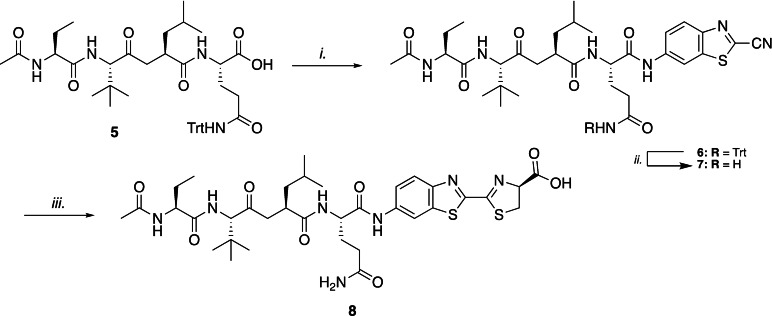
Three‐step aLuc conjugation with peptide **5** to afford luminescent probe **8**. Reagents and conditions: i. 6‐ABTC (1.4 equiv), *N*‐methylmorpholine (3.0 equiv), isobutyl chloroformate (1.8 equiv), THF. ii. TFA, TIS, H_2_O (95/2.5/2.5, v/v). iii. d‐cysteine⋅HCl (1.1 equiv), K_2_CO_3_ (1.1 equiv), PBS (pH=7.2), RP‐HPLC, 3 % over three steps.

The peptide Ac‐Abu‐Leu‐Tle‐Gln(Trt)‐COOH (**5**) was synthesized using standard SPPS Fmoc chemistry on a 2‐chlorotrityl chloride resin. After cleavage under mild conditions (TFE : HOAc : DCM, 1 : 1 : 3, v/v), the protected peptide was coupled to aLuc in a three‐step synthesis procedure (Scheme 2). Since the thiazoline ring in luciferin is known to be susceptible to oxidation, we first introduced the more stable 6‐ABTC moiety by coupling it to protected peptide **5**. The carboxylic acid of **5** was activated with isobutyl chloroformate in the presence of *N*‐methylmorpholine to form the mixed anhydride, which was followed by the addition of 6‐ABTC (**4)** to afford intermediate **6**. With this procedure undesired epimerization of the C‐terminal amino acid could be minimized.^[21]^ Hereafter, the trityl group was removed using trifluoroacetic acid in the presence of triisopropylsilane and water to afford **7**. Finally, reaction with d‐cysteine in PBS afforded the desired caged aLuc probe **8**, which was purified using reversed phase HPLC.

Recombinant M^Pro^ protein was expressed to enable assay development. To this end, the gene encoding for SARS‐CoV‐2‐19 M^Pro^ from isolate Wuhan‐Hu‐1 was cloned in frame with a C‐terminal His_6_‐tag in pET28a, expressed in *E. coli* and purified (see Experimental Section). The N‐terminus was flanked with a short peptide sequence encoding the natural cleavage site for M^Pro^ resulting in a native N‐terminus after expression.

**Figure 2:**
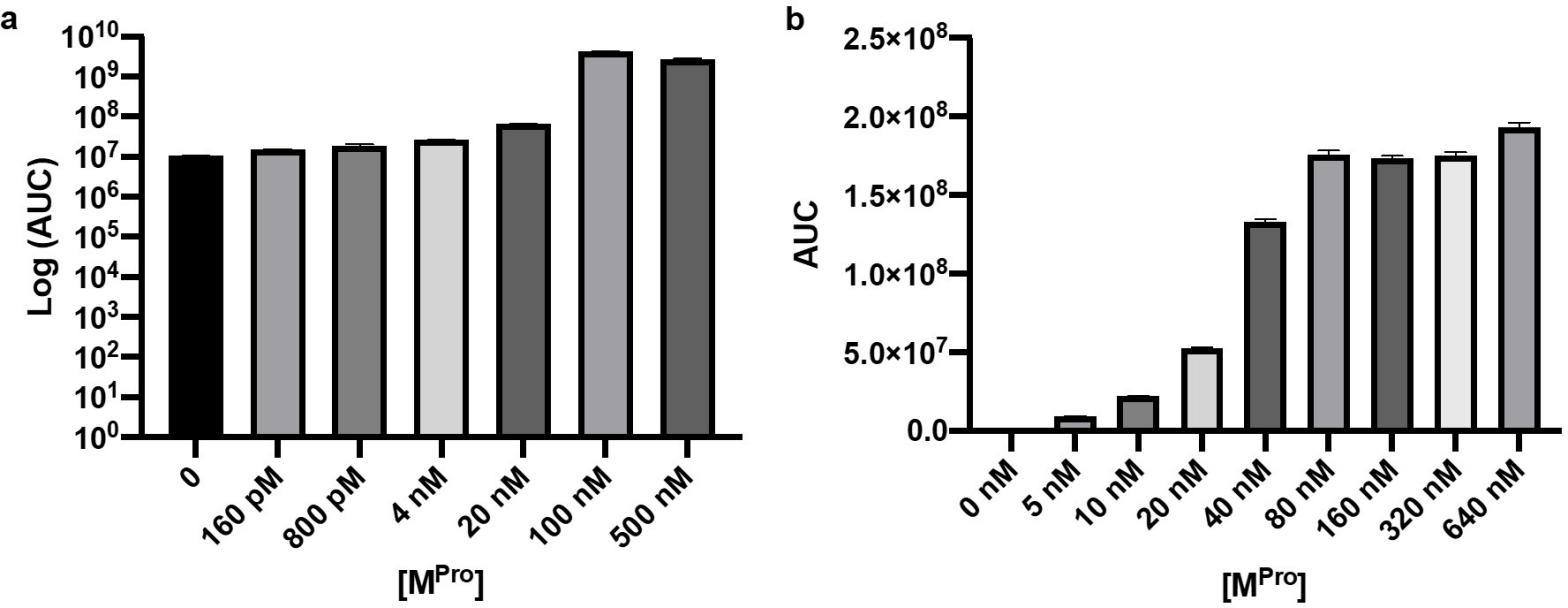
Detection limit determination experiments for SARS‐CoV‐2 M^Pro^ with a constant concentration of probe **8** (20 μM). a. Lower limit of detection M^Pro^ titration from 160 pM–500 nM, significant signal in respect to the blank (no M^Pro^ added) was obtained from 4 nM. b. Linear M^Pro^ titration from 5–640 nM, significant signal in respect to the blank (no M^Pro^ added) was obtained from 5 nM.

With substrate **8** and recombinant M^Pro^ in hands we set out to determine the sensitivity of the assay. Substrate **8** was incubated for 1 h. at 37 °C in the presence of different concentrations M^Pro^, followed by the addition of the luminescent detection mix (ATP, MgCl_2_ and Ultra‐Glo™ rLuciferase, Promega) to generate a proportional light signal *via* the conversion of the released aLuc. Protease cleavage is known to be the rate‐determining step as compared to luciferase catalysis in this secondary read‐out.[^15^, ^22^] The pre‐incubation of substrate with protease for 1 h. gave satisfactory signal‐to‐background sensitivity due to the accumulation of aLuc product prior to addition of luciferase. The luminescence was recorded at room temperature using a luminometer and afforded the data as depicted in Figure 2. Initial titration experiments of M^Pro^ revealed that our lower limit of detection was 4 nM (Figure 2, a). Hereafter, we evaluated our assay within 0–640 nM to find the linear range and highest limit of detection of our assay (Figure 2, b). The highest limit of detection was found to be 80 nM and our luminescent signal was linear within the 0–80 nM range (see Figure S12)

**Figure 3:**
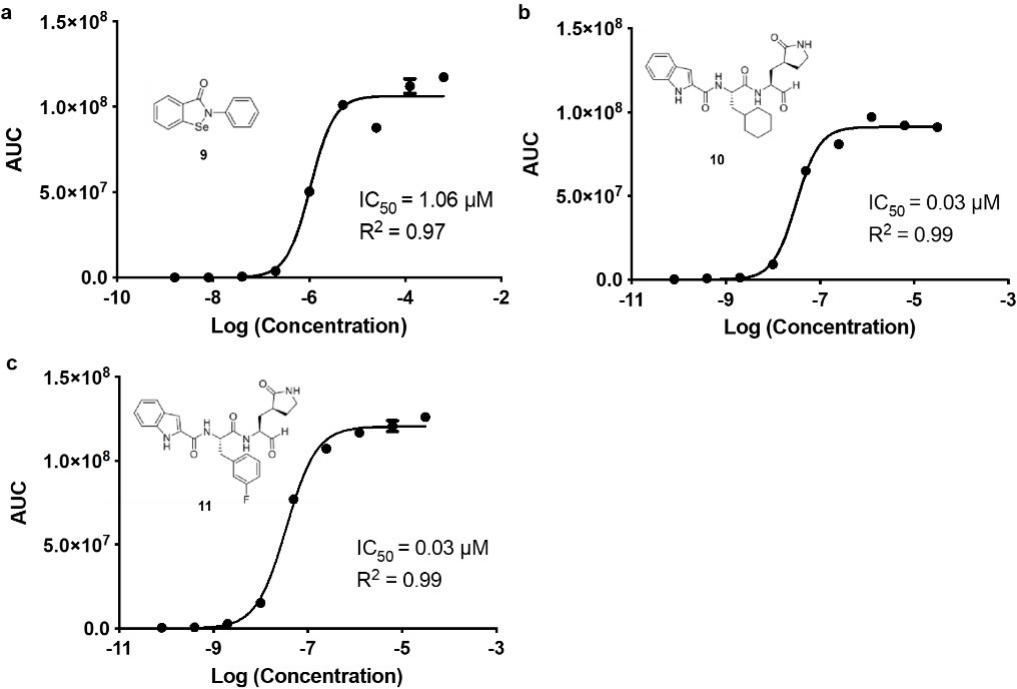
Inhibition assays **a**–**c** for SARS‐CoV‐2 M^Pro^, measured at decreasing inhibitor concentrations. a. Ebselen (**9**), b. RU‐02‐005 (**10**), c. RU‐02‐006 (**11**). All datapoints were measured in duplicate (error bars indicated).

After validation, we examined the potential of our assay to function as a screening platform for the detection of potential M^Pro^ inhibitors. To validate that our luminescent based signal was enzyme dependent, we incubated substrate **8** and M^Pro^ for 1 h. at 37 °C with varying concentrations of Ebselen (**9**), RU‐02‐005 (**10**) and RU‐02‐006 (**11**), which all three have recently been reported to be potent M^Pro^ inhibitors and their expected mechanistic mode of action were described.[^23^, ^24^] For M^Pro^ a constant concentration of 30 nM was used since this fitted within the linear range of our detection. After addition of the detection mix, the luminescence was recorded at room temperature, providing the results depicted in Figure 3. The IC_50_ values were afforded after AUC analysis of all duplicate measurements and the corresponding 95 % confidence interval (CI) were determined (Table 1).

**Table 1:** IC_50_ values determined in this study.

Inhibitor	IC_50_	95 % CI^[a]^	Literature
9	1.06 μM	0.58–1.92 μM	0.67 μM^[24]^
10	0.03 μM	0.02–0.04 μM	0.05 μM^[23]^
11	0.03 μM	0.03–0.05 μM	0.04 μM^[23]^

[a] 95 % confidence interval (asymptotic).

The determined IC_50_ value of **9** was found to be 1.06 μM, similar as to the value determined by Jin and co‐workers.^[24]^ (Figure 3, a). For both compound **10** and **11** a value of 0.03 μM was found as analogous to those from the literature (Figure 3, b and c).^[23]^

## Conclusion

In conclusion, we developed a luminescent assay for the detection of potential inhibitors of the viral chymotrypsin‐like protease M^Pro^ from SARS‐CoV‐2. Our luminescent probe Ac‐Abu‐Tle‐Gln‐aLuc (**8**) contains a specific cleavage site for M^Pro^ and after proteolytic activity the aLuc releases photons in the presence of luciferase. The detection limit of our assay was found to be within 4–80 nM and our linear signal was shown to be M^Pro^ dependent. We demonstrated that our luminescent assay could be used to screen potential M^Pro^ inhibitors. For three potent inhibitors Ebselen (**9**), RU‐02‐005 (**10**) and RU‐02‐006 (**11**) the IC_50_ values were determined to be in the μM scale. The reported assay unravels new avenues for the rapid screening of large numbers of SARS‐CoV‐2 M^Pro^ inhibitors and could potentially be used for diagnostic purposes. Besides, the use of luminescence renders our approach suitable for miniaturization and potential high throughput screening applications.

## Experimental Section

**Recombinant protein production**: An *Escherichia coli* (*E. coli*) codon optimized sequence encoding SARS‐CoV‐2 M^Pro^ (ORF1ab polyprotein residues 3264–3569, GenBank code: MN908947.3) was synthesized by IDT integrated DNA technologies (Coralville, Iowa, USA). The gene was amplified and extended with forward primer 5′‐CGCGGATCCT CGGCAGTGCT GCAATCGGGG TTTCGCAAAAT‐3′ and reverse primer 5′‐CCGCTCGAGC TGAAACGTGA CACCGCTACA‐3′ which includes restriction sites for cloning into a pET‐28a vector. The forward primer includes a M^Pro^ cleavage site (SAVLQ↓SGFRK; arrow indicates the cleavage site), the C‐terminus is in frame with the His_6_‐tag on pET‐28a. The PCR product was digested with BamHI and XhoI, ligated in pET‐28a digested with the same restriction enzymes and transformed in *E. coli* TOP10 cells by heat shock. Single‐colony transformants were selected on LB‐agar plates containing 50 μg/mL kanamycin and verified using Sanger sequencing. For expression the plasmids were transformed into *E. coli* BL21(DE3) cells. SARS‐CoV‐2 M^Pro^ gene expression was performed in 1 L LB supplemented with 50 μg/mL kanamycin, inoculated with 25 mL pre‐culture grown overnight at 37 °C. At an OD600=0.8, the temperature was decreased to 18 °C and gene expression was induced with 0.1 mM isopropyl‐β‐d‐1‐thio‐galactopyranoside (IPTG) for 20 h. Cells were harvested by centrifugation at 5,000 *g* at 4 °C for 20 minutes in a JA‐10 rotor (Beckman Coulter Avanti J26S XP). The pellet was resuspended in lysis buffer (20 mM Tris, 150 mM NaCl, pH 7.8) and lysed by sonication on ice (8×1.5 min). After precipitation of the insoluble debris by centrifugation at 15,000 rpm at 4 °C for 40 minutes in a JA‐25.50 rotor, the cleared supernatant was loaded onto a HisTrap FF column (Cytiva) equilibrated with lysis buffer. The column was washed with wash buffer (20 mM Tris, 150 mM NaCl, 50 mM imidazole, pH 7.8) followed by elution with (20 mM Tris, 150 mM NaCl, 500 mM imidazole, pH 7.8). The fractions containing protein of target mass were pooled and dialyzed with dialysis buffer (20 mM Tris, 1 mM DTT, pH 8.0) at 4 °C, overnight. The product was further purified using a HiTrap Q FF column (GE Healthcare) equilibrated with the same buffer. The protein was eluted with elution buffer (20 mM Tris, 1 M NaCl, 1 mM DTT, pH 8.0) applying a linear gradient ranging from 0 to 500 mM NaCl (20 column volumes buffer). The fractions containing protein of target mass were pooled and subjected to dialysis (20 mM Tris, 150 mM NaCl, 1 mM EDTA, 1 mM DTT, pH 7.8).

**M^Pro^ detection limit assay**: Varying concentrations of SARS‐CoV‐2 M^Pro^ were added to a well containing Ac‐Abu‐Tle‐Leu‐Gln‐aLuc (final conc. 20 μM) and reaction buffer (25 mm HEPES buffer, 125 mM NaCl pH 7.4) with a final volume of 25 μL. After incubation for 1 h. at 37 °C, 5 μL of the detection mix was added containing Ultra‐Glo™ rLuciferase (Promega) (final conc. 0.69 mg/mL), MgCl_2_ (final conc. 8.3 mM) and ATP (final conc. 330 μM). The luminescence was recorded in relative light units (RLU) for 40 minutes at 20 °C with a M3 plate reader (Molecular Devices, San Jose, CA, USA). The total luminescence (AUC) was plotted as a function of M^Pro^ concentration GraphPad Prism (version 9.0).

**M^Pro^ linear titration assay**: Varying concentrations of SARS‐CoV‐2 M^Pro^ were added to a well containing Ac‐Abu‐Tle‐Leu‐Gln‐aLuc (final conc. 20 μM) and reaction buffer (20 mm HEPES buffer, 125 mM NaCl pH 7.4) with a final volume of 50 μL. After incubation for 1 h. at 37 °C, 10 μL of the detection mix was added containing Ultra‐Glo™ rLuciferase (Promega) (final conc. 0.14 mg/mL), MgCl_2_ (final conc. 1.7 mM) and ATP (final conc. 66 μM). The luminescence was recorded in relative light units (RLU) for 40 minutes at 20 °C with a M3 plate reader (Molecular Devices, San Jose, CA, USA). The total luminescence (AUC) was plotted as a function of M^Pro^ concentration GraphPad Prism (version 9.0). For the linear regression plot the luminescence was normalized for the signal of the blank (no M^Pro^ added) (Figure 2, c).

**M^Pro^ inhibition assay**: SARS‐CoV‐2 M^Pro^ (final conc. 30 nM) was added to a well containing Ac‐Abu‐Tle‐Leu‐Gln‐aLuc (final conc. 20 μM) and varying concentration of inhibitor in reaction buffer (25 mm HEPES buffer, 125 mM NaCl pH 7.4) with a final volume of 50 μL. After incubation for 1 h. at 37 °C, 10 μL of the detection mix was added containing Ultra‐Glo™ rLuciferase (Promega) (final conc. 0.14 mg/mL), MgCl_2_ (final conc. 1.7 mM) and ATP (final conc 66 μM). Luminescence was recorded for 40 minutes at 20 °C with a M3 plate reader (Molecular Devices, San Jose, CA, USA). Luminescence was plotted as a function of inhibitor concentration in GraphPad Prism (version 9.0, data fitted with non‐linear regression, n=2), the IC_50_ values were determined using the AUC over the total 40 minutes of each concentration.

## Conflict of interest

The authors declare no conflict interest.

1

## Supporting information

As a service to our authors and readers, this journal provides supporting information supplied by the authors. Such materials are peer reviewed and may be re‐organized for online delivery, but are not copy‐edited or typeset. Technical support issues arising from supporting information (other than missing files) should be addressed to the authors.

Supporting Information

## Data Availability

The data that support the findings of this study are available from the corresponding author upon reasonable request.
