# Supplementary material for: Luminescent Assay for the Screening of SARS‐CoV‐2 MPro Inhibitors
Source: Chembiochem. 2022 Jun 14;23(15):e202200190. doi: 10.1002/cbic.202200190 (PMC9401586; doi:10.1002/cbic.202200190)
Supplement: Supplementary file 1 — Supporting Information [file CBIC-23-0-s001.pdf]

ChemBioChem

Supporting Information

Luminescent Assay for the Screening of SARS-CoV-2 M^{Pro} Inhibitors

Daan Sondag[+], Jona Merx[+], Emiel Rossing, Thomas J. Boltje, Dennis W. P. M. Löwik, Frank H. T. Nelissen, Mark van Geffen, Cornelis van ’t Veer, Waander L. van Heerde, and Floris P. J. T. Rutjes*

Table of Contents

1. General S2

2. *E. coli* codon optimized sequence of M^{Pro} S3

3. 6-Aminobenzo[*d*]thiazole-2-carbonitrille synthesis S4

4. Peptide synthesis S6

5. NMR spectra S8

6. LCMS spectra S12

7. Inhibition experiments raw data S16

8. Linear titration data S19

9. References S20

1. General

NMR spectra were recorded on a Bruker Avance III 400 MHz or a Bruker 500 MHz spectrometer and the compounds were assigned using ^{1}H NMR, ^{13}C NMR, COSY, HSQCED and HMBC spectra. Chemical shifts were reported in parts per million (ppm.) relative to reference (CD_3OD: ^{1}H: 3.31 ppm. and ^{13}C 49.00 ppm; $CDCl_3$: ^{1}H: 7.26 ppm. and ^{13}C 77.16 ppm.) NMR data are presented in the following way: chemical shift, multiplicity (s = singlet, bs = broad singlet, d = doublet, t = triplet, dd = doublet of doublets, ddd = doublet of doublet of doublets, dtd = doublet of triplet of doublets h = heptet, m = multiplet and/or multiple resonances) and coupling constants *J* in Hz. The peptide was synthesized using Fmoc solid-phase peptide (SPPS) chemistry on a 2-chlorotrityl chloride resin (2CTC). On resin peptide couplings and deprotections were monitored using Kaiser tests. Mass spectra were recorded on a JEOL AccuTOF CS JMS-T100CS (ESI) mass spectrometer and LCMS analysis was done on an ISQ™ EM Single Quadrupole Mass Spectrometer (Thermo Fisher). Automatic flash column chromatography was executed on a Biotage Isolera Spektra One using SNAP or Silicycle cartridges (Biotage, 30–100 µm, 60Å) 4–50 g. Preperative HPLC was performed on a Phenomenex® Gemini-NX 3u C18 110A reversed-phase column (150 x 21.2 mm) using gradient elution with a constant flow of 10 mL/min at 30 °C. MiliQ (0.1% TFA) and CH_3CN (0.1% TFA) were used as the solvents. The pure fractions containing product were combined and lyophilized overnight to yield the target compounds. Reactions under protective atmosphere were performed under positive Ar./N_2 flow using flame-dried glassware. The inhibitors RU-02-005 and RU-02-006 were supplied in-house and Ebselen was bought from a commercial supplier (TCI Europe N.V.).

2. *E. coli* codon optimized sequence of M^{Pro} from SARS-CoV-2 as used in this study

TCGGGGTTTCGCAAAATGGCGTTTCCGAGCGGAAAAGTAGAAGGCTGCATGGTTCAGGTG
ACTTGTGGTACGACCACGCTTAACGGGTTGTGGTTAGATGATGTGGTCTATTGTCCGCGTC
ATGTCATCTGCACTTCCGAGGATATGCTGAACCCGAACTACGAAGATCTGCTTATCCGGAA
AAGCAACCACAACTTCCTCGTTCAGGCAGGGAATGTACAGTTGCGTGTGATTGGGCATTCG
ATGCAGAATTGCGTGCTCAAACTGAAAGTCGATACGGCGAATCCCAAAACCCCGAAATACA
AGTTTGTTCGCATTCAGCCAGGACAGACCTTTAGCGTTTTGGCCTGTTATAACGGTAGTCC
GAGTGGCGTCTATCAATGCGCCATGCGTCCGAATTTCACCATCAAGGGTAGCTTCCTCAAT
GGTTCGTGTGGTTCCGTAGGCTTTAACATCGATTACGACTGCGTTTCGTTCTGCTACATGCA
CCATATGGAACTGCCTACCGGTGTTCATGCTGGGACAGACTTAGAAGGCAACTTTTATGGA
CCCTTTGTCGACCGCCAGACAGCGCAAGCAGCAGGCACAGATACCACGATTACCGTGAAC
GTCTTAGCGTGGCTGTATGCGGCAGTGATCAATGGCGATCGCTGGTTCCTTAACCGCTTTA
CGACTACCCTGAATGACTTCAATTTAGTTGCCATGAAGTACAACTATGAACCACTGACCCAA
GATCACGTAGACATTCTGGGTCCTCTGTCTGCTCAAACGGGCATTGCTGTGCTGGACATGT
GTGCGAGCCTGAAAGAGCTGCTGCAGAACGGTATGAATGGCCGCACTATTCTGGGTTCAG
CCTTGCTGGAAGATGAGTTCACCCCGTTTGATGTGGTGCGTCAATGTAGCGGTGTCACGTT
TCAG

3. 6-Aminobenzo[*d*]thiazole-2-carbonitrile synthesis[1]

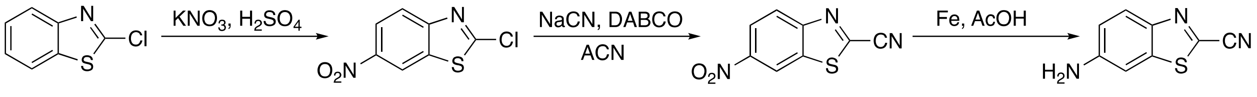

1 **2** **3** **4**

Scheme S1: *Three-step synthesis of 6-aminobenzo[d]thiazole-2-carbonitrile from 2-chlorobenzothiazole.*

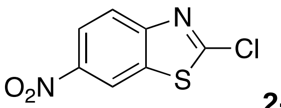

2-Chloro-6-nitrobenzo[*d*]thiazole (2): 2-Chlorobenzo[*d*]thiazole (**1**, 15.0 g, 88.43 mmol) was added portion wise to H_2SO_4 (98%, 90 mL) on an ice bath. KNO_3 (9.834 g, 97.27 mmol) was added portion wise and the reaction was stirred at 0 °C for 30 min. The reaction mixture was allowed to warm up to rt and stirred for 18 h. at this temperature. The mixture was poured on ice water (300 mL) and the formed precipitate was collected by filtration. The crude product was rinsed with ice cold water and aqueous $NaHCO_3$ until pH >7. The product was dried under reduced pressure overnight and recrystallized from EtOH (650 mL) to afford **2** (16.81 g, 89%) as an off-white solid. $\mathbf{R_f}$ = 0.60 (EtOAc/n-heptane, 1:4 v/v). **^{1}H NMR** (500 MHz, $CDCl_3$) δ 8.75 (d, *J* = 2.3 Hz, 1H), 8.38 (dd, *J* = 8.9, 2.3 Hz, 1H), 8.07 (d, *J* = 9.0 Hz, 1H).**^{13}C NMR** (126 MHz, $CDCl_3$) δ 158.9, 154.9, 136.6, 123.5, 122.4, 117.8.

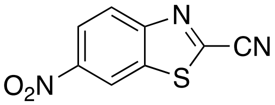

6-Nitrobenzo[*d*]thiazole-2-carbonitrile (3): 2-Chloro-6-nitrobenzo[*d*]-thiazole (**2**, 10.78 g, 50.23 mmol) was dissolved in ACN (1000 mL) and DABCO (845.2 mg, 7.53 mmol) was added. NaCN (2.78 g, 56.83 mmol) was dissolved in water (100 mL) and added dropwise to the stirred reaction mixture. After 24 h. the reaction mixture was quenched with aqueous iron(iii) chloride hexahydrate ($FeCl_3.6H_2O$) (0.3 M, 50 mL) and diluted with water (350 mL). The reaction mixture was extracted with EtOAc (3x 400 mL) and the combined organic layers were washed with brine (100 mL), dried with $MgSO_4$ and concentrated *in vacuo*. The crude product was loaded on a silica plug and flushed with $CHCl_3$ (2000 mL), concentrated and dried *in vacuo* to afford **3** (8.30 g, 81%) as a yellow solid. $\mathbf{R_f}$ = 0.33 ($CHCl_3$). **^{1}H NMR** (500 MHz, $CDCl_3$) δ 8.95 (d, *J* = 2.2 Hz, 1H), 8.52 (dd, *J* = 9.1, 2.2 Hz, 1H), 8.38 (d, *J* = 9.1 Hz, 1H). **^{13}C NMR** (126 MHz, $CDCl_3$) δ 155.5, 147.4, 141.9, 135.7 126.2, 123.2, 118.7, 112.1.

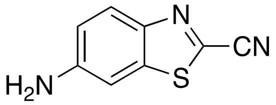

6-Aminobenzo[*d*]thiazole-2-carbonitrile (4) 6-Nitrobenzo[*d*]-thiazole-2-carbonitrile (**3**, 7.70 g, 37.53 mmol) was suspended in AcOH (700 mL). Iron dust (104.80 g, 1.88 mol) was added and the reaction was stirred for 24 h. The reaction was diluted with water (1400 mL) and filtered over Celite. The aqueous solution was extracted with EtOAc (3x 700 mL) and washed with brine (400 mL). The crude product was loaded on a silica plug and flushed with $CHCl_3$ (2000 mL), concentrated and dried *in vacuo* to afford **4** (3.45 g, 53%) as a yellow solid. **Rf** = 0.26 (DCM). **^{1}H NMR** (500 MHz, $CDCl_3$) δ 7.95 (d, *J* = 8.9 Hz, 1H), 7.08 (d, *J* = 2.2 Hz, 1H), 6.95 (dd, *J* = 8.9, 2.3 Hz, 1H). **^{13}C NMR** (126 MHz, $CDCl_3$) δ 147.6, 145.5, 138.0, 131.0, 125.9, 117.6, 103.8, 77.2. **HRMS** (m/z): $[M + H]^+$ calcd. for $C_8H_5N_3S$: 176.0282, found 176.0294.

4. Peptide synthesis

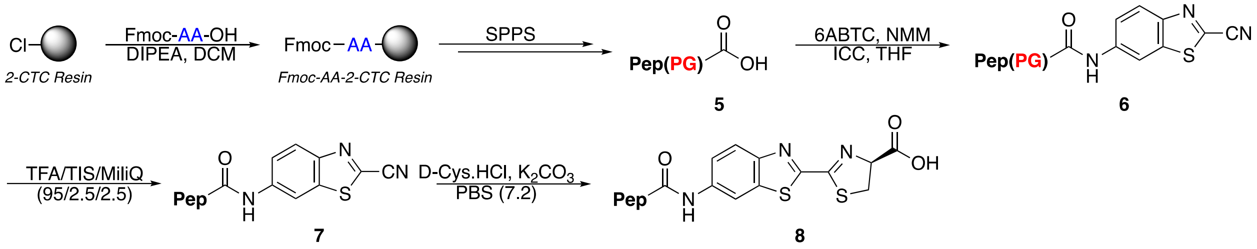

Scheme S2: *Synthesis of aminoluciferin conjugated peptides.*

Loading of 2-CTC resin (Fmoc-Gln(Trt)-2-CTC):

Fmoc-AA-OH (2.0 equiv) was dissolved in DCM (10 mL/g resin), vortexed and added to 2-CTC resin. DIPEA (2.1 equiv) was added and the resin was agitated for 1 h. MeOH (HPLC grade, 0.8 mL/g resin) and DIPEA (1.0 equiv) were added and the resin was agitated for 15 min. The resin was filtered and washed with DCM (3x), DMF (3x), MeOH (3x), dried *in vacuo* overnight and the loading was determined by weight gain.

SPPS of the protected peptides:

The Fmoc-Gln(Trt)-2-CTC resin was swollen for 20 min in DMF, hereafter the Fmoc was deprotected with 20% piperidine in DMF (2 x 6 min). All amino acids (3.0 equiv) were pre-mixed with HATU (2.9 equiv), DIPEA (6.0 equiv) and subsequently added to the resin and agitated for 1 h. The resin was washed with DMF (3x) and capped with pyridine (20 equiv) and Ac_2O (20 equiv) for 10 min. The resin was washed with DMF (3x) and this cycle was repeated until the desired sequence was obtained. The protected peptide was cleaved with TFE/HOAc/DCM (1:1:3, v/v) for 1 h. after which the resin was washed with DCM (3x) and the product was concentrated and lyophilized in water overnight to obtain the desired protected peptide. The obtained crude peptide was directly used in the next steps without further purification.

Aminoluciferin conjugation[2]:

The protected peptide (1.0 equiv) was dissolved in dry THF (5 mL / 100 mg) in a flame-dried flask. The solution was cooled to -10 °C, 4-methylmorfolin (3.0 equiv) and isobutyl carbonchloridate (1.8 equiv) were added and the reaction was stirred for 1 h. at this temperature. 6-aminobenzo[*d*]thiazole-2-carbonitrille (1.4 equiv) in THF 2 mL / 30 mg) was added and the reaction was allowed to warm up to rt and stirred overnight. The reaction mixture was concentrated, dissolved in EtOAc and washed with water (2x) and brine. The aqueous phase was extracted with EtOAc and the combined organic layers were concentrated *in vacuo*. The crude product was

purified with silica gel column chromatography (60 → 100 % EtOAc in *n*-heptane) to afford the protected peptide-6-aminobenzo[*d*]thiazole-2-carbonitrille. The conjugated peptide was dissolved in TFA/TIS/H_2O (95/2.5/2.5, v/v) and stirred for 3 h. after which it was concentrated *in vacuo* and lyophilized in water. The product was dissolved in PBS (pH= 7.2) and D-cysteine.HCl (1.1 equiv) in H_2O and K_2CO_3 (1.1 equiv) were added and the reaction was stirred in the dark for 1 h. The reaction mixture was concentrated *in vacuo* and the crude product was purified using reversed-phase preparative HPLC (0→100 % ACN (0.1% TFA) in MiliQ (0.1% TFA)) and lyophilized to afford the final desired peptide **8**.

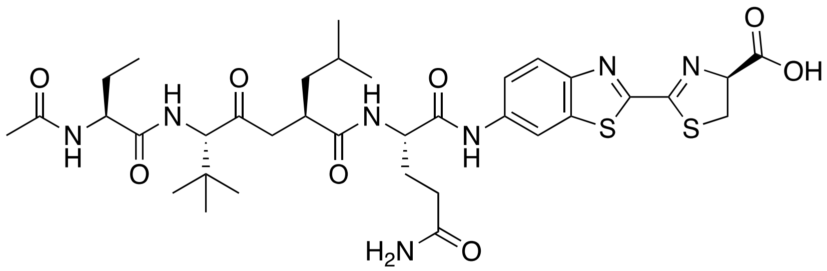

Ac-Abu-Tle-Leu-Gln-aLuc (8): Was prepared according to the general peptide synthesis procedure and obtained as a yellow solid (11.8 mg, 3% over 3 steps). **^{1}H NMR** (500 MHz, CD_3OD) δ 8.48 (d, *J* = 2.2 Hz, 1H), 7.99 (d, *J* = 8.8 Hz, 1H), 7.65 (dd, *J* = 9.0, 2.1 Hz, 1H), 5.45 – 5.40 (m, 1H), 4.53 (dd, *J* = 9.2, 5.1 Hz, 1H), 4.45 (dd, *J* = 9.2, 5.8 Hz, 1H), 4.32 – 4.28 (m, 2H), 3.84 – 3.74 (m, 2H), 2.40 (t, *J* = 7.5 Hz, 2H), 2.22 (ddd, *J* = 13.0, 7.6, 3.8 Hz, 1H), 2.11 – 2.06 (m, 1H), 2.00 (s, 3H), 1.83 – 1.75 (m, 2H), 1.70 – 1.60 (m, 3H), 1.01 (s, 9H), 0.96 – 0.91 (m, 9H). **^{13}C NMR** (100 MHz, CD_3OD) δ 174.7, 174.4, 173.7, 173.4, 172.8, 172.2, 172.1, 167.6, 161.2, 150.7, 139.0, 125.2, 121.4, 113.5, 79.4, 62.3, 56.5, 55.0, 53.3, 41.5, 35.9, 35.3, 32.5, 28.9, 27.2, 25.8, 23.4, 22.4, 22.0, 10.7. **HRMS** (m/z): $[M + H]^+$ calcd. for $C_{34}H_{48}N_8O_8S_2$: 761.3114, found 761.3115. **Purity** (LCMS): 94%.

5. NMR spectra

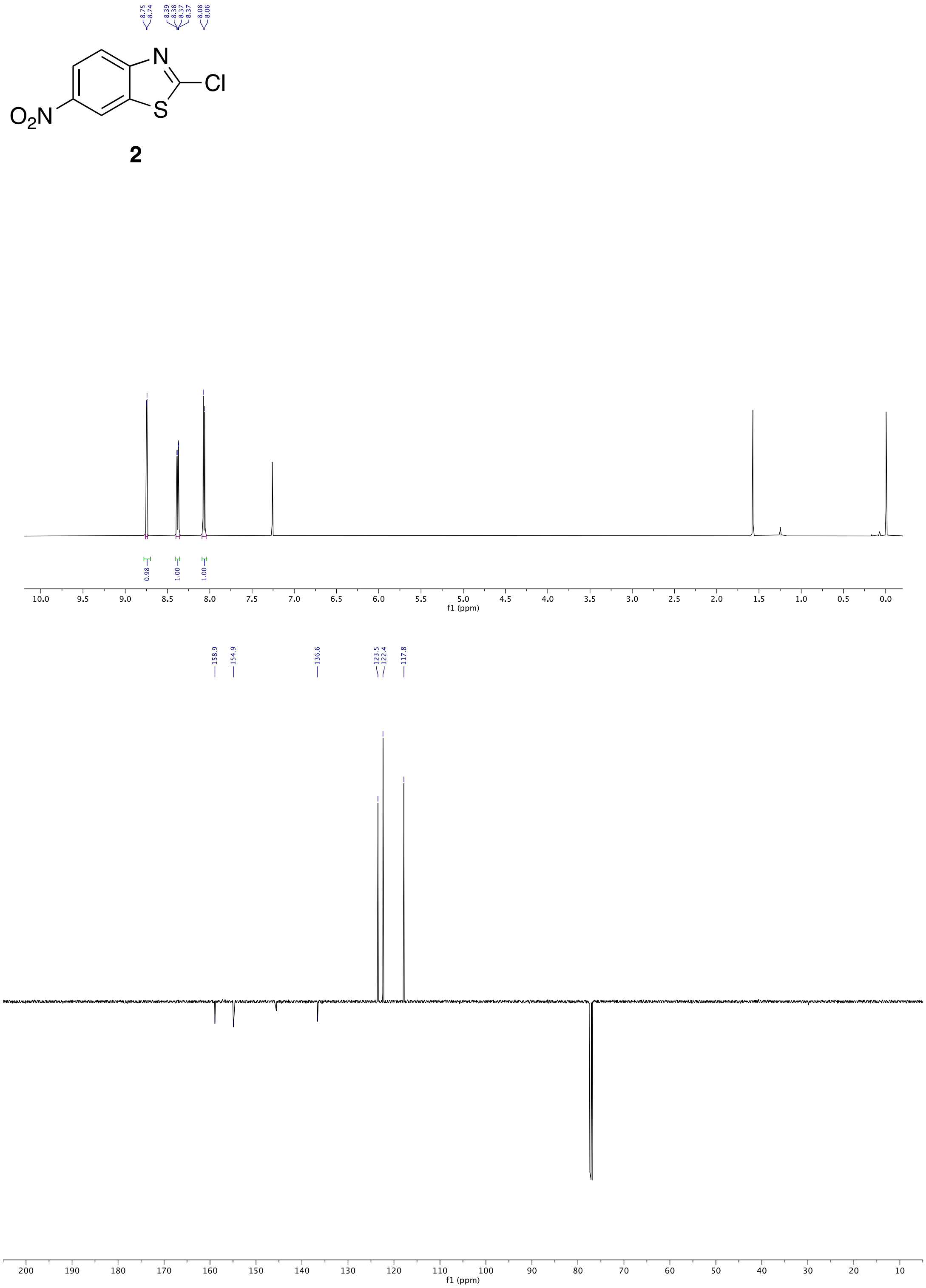

Figure S1: *^{1}H and ^{13}C NMR spectrum of compound* ***2.***

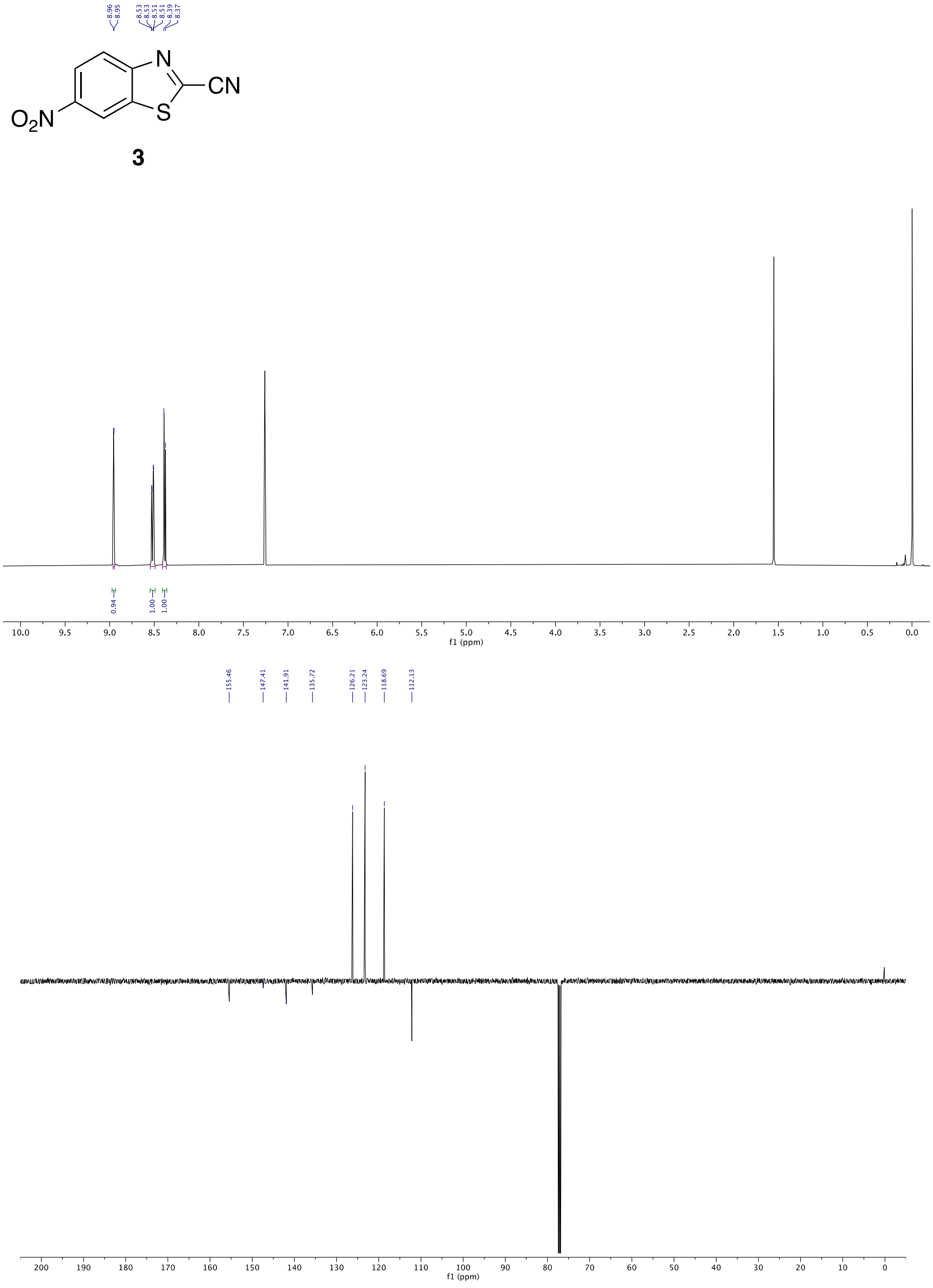

Figure S2: *^{1}H and ^{13}C NMR spectrum of compound* ***3.***

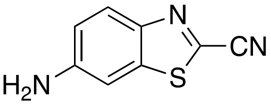

4

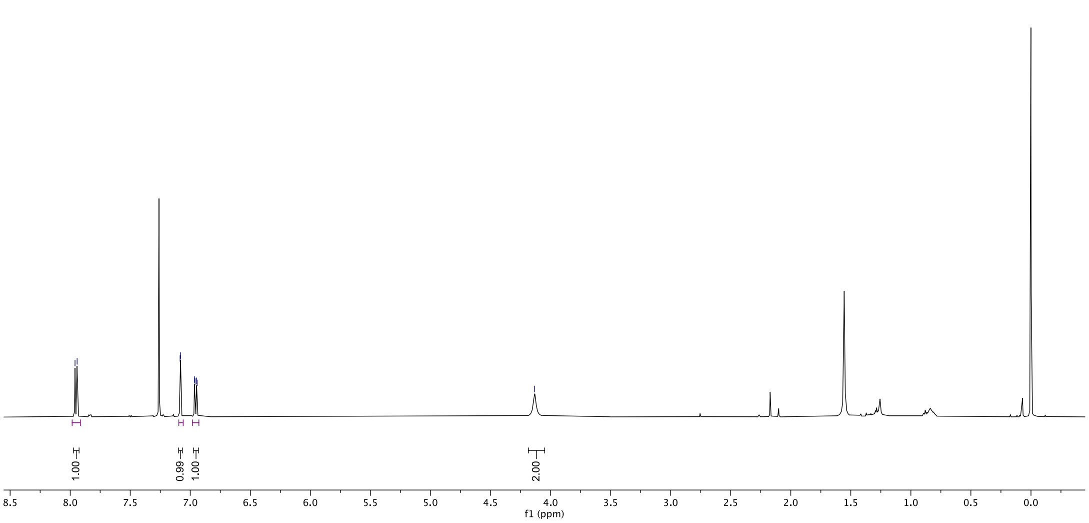

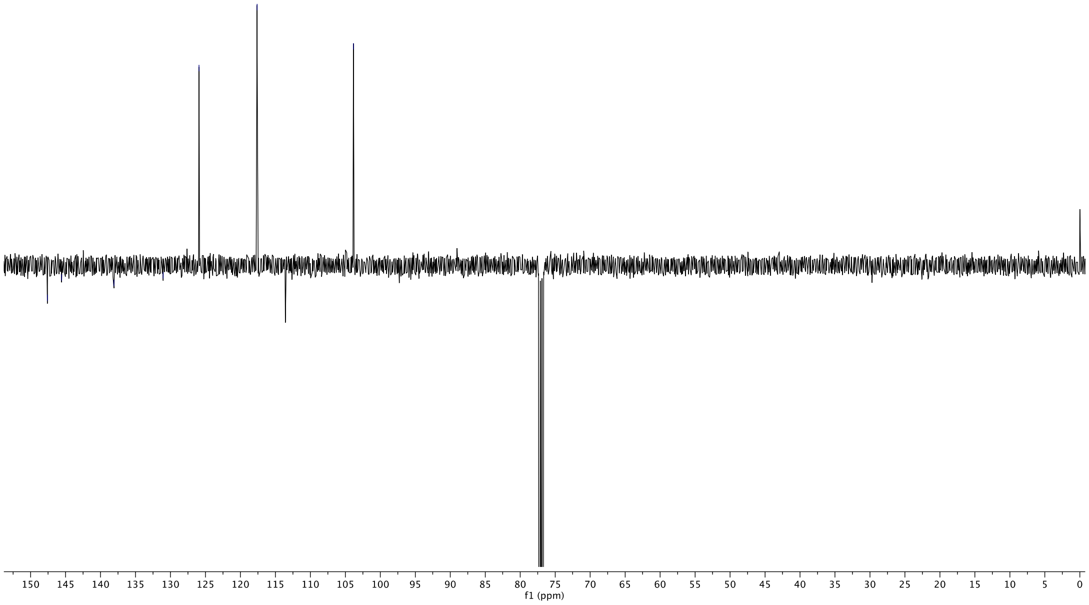

Figure S3: *^{1}H and ^{13}C NMR spectrum of compound **4**.*

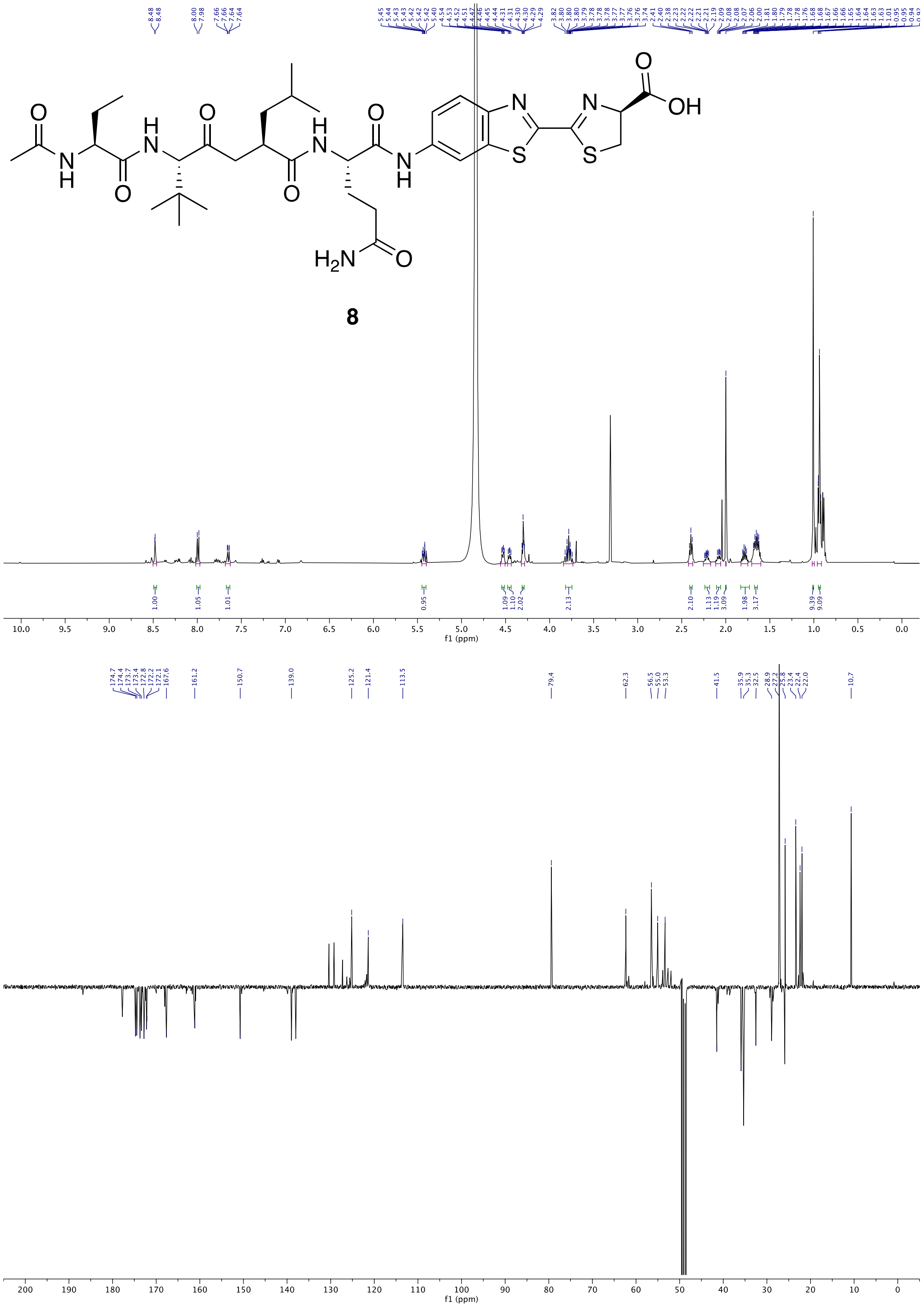

Figure S4: *¹H and ¹³C NMR spectrum of compound **8**.*

6. LCMS spectra

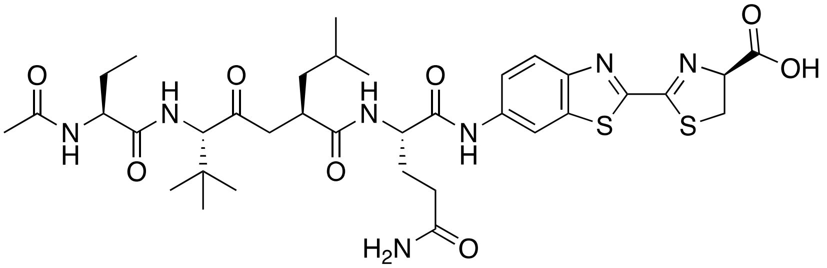

8

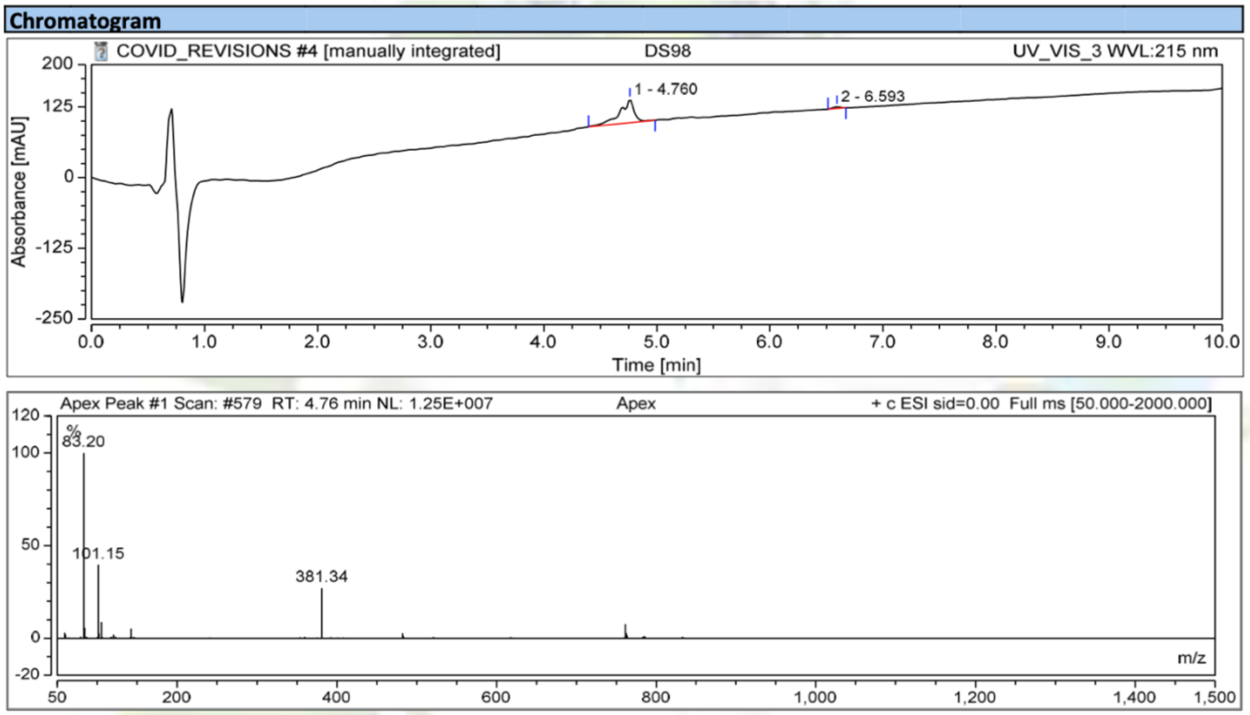

Figure S5: *LCMS spectrum of compound* ***8****.*

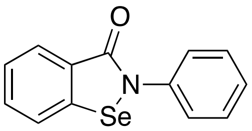

9

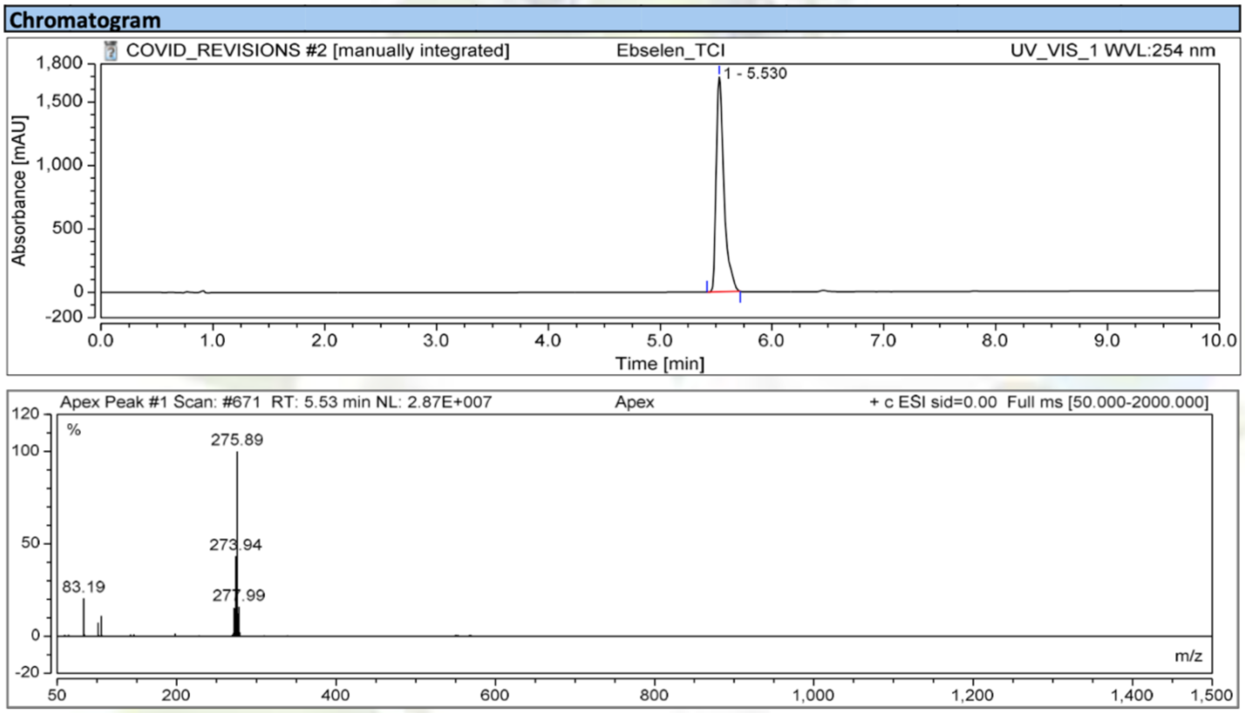

Figure S6: *LCMS spectrum of compound* ***9***.

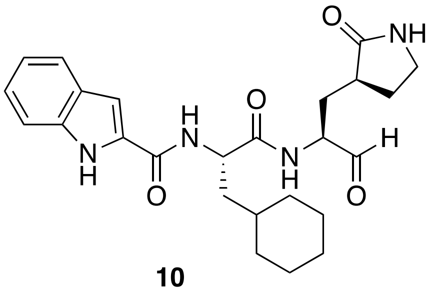

10

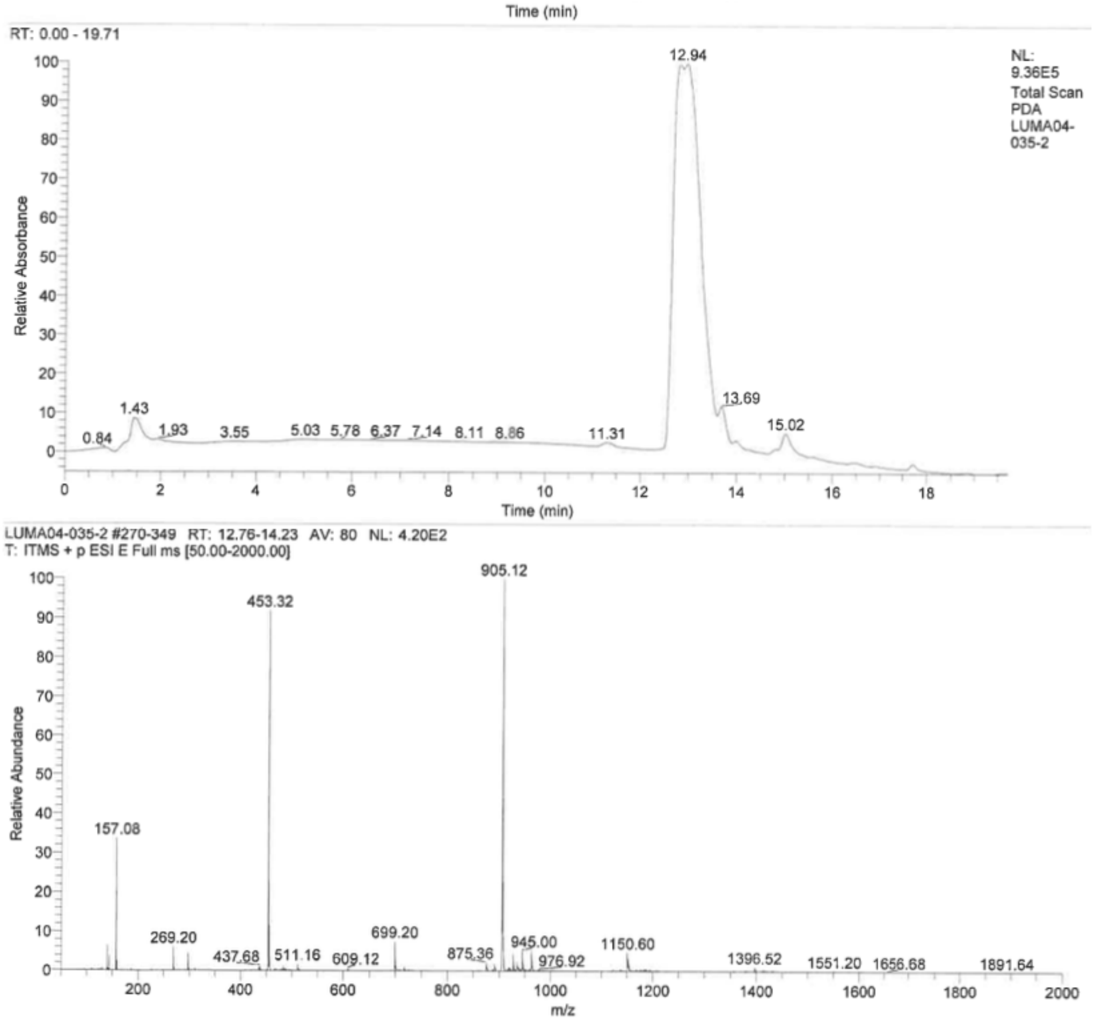

Figure S7: *LCMS spectrum of compound* ***10.***

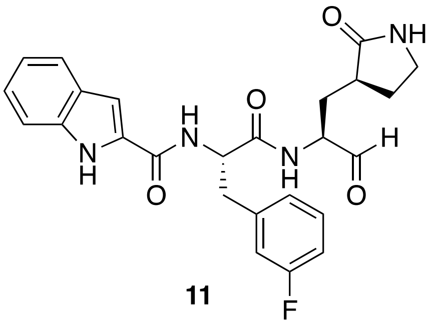

11

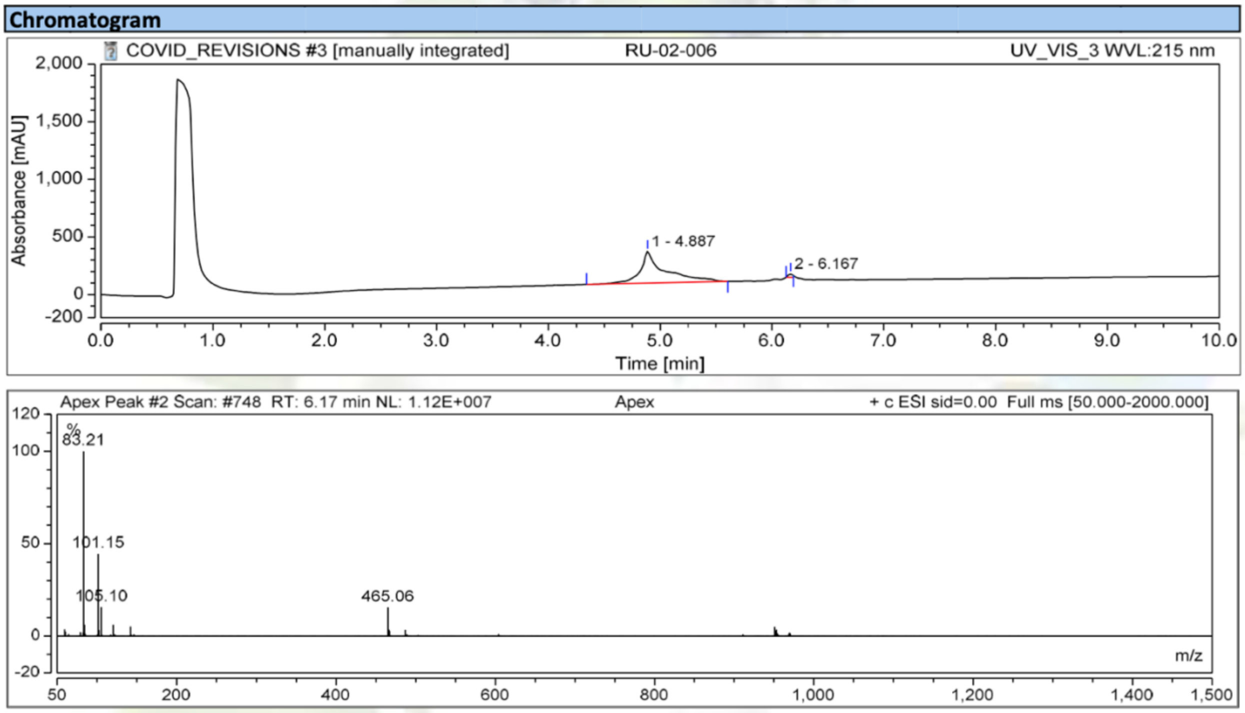

Figure S8: *LCMS purity spectrum of compound* ***11.***

7. Inhibition experiments raw data

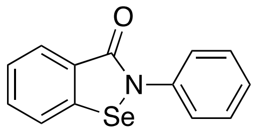

9

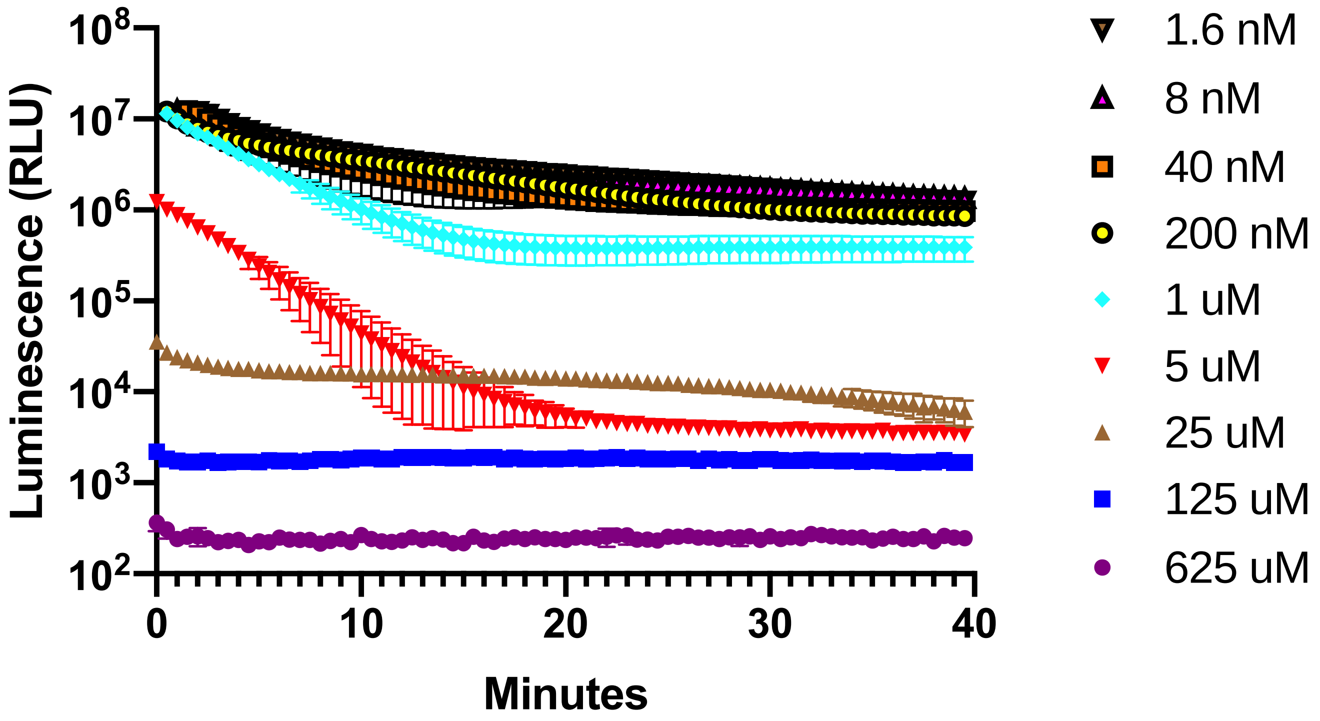

Figure S9: *Raw data of the inhibition experiments for the IC_{50} assay of compound* ***9.***

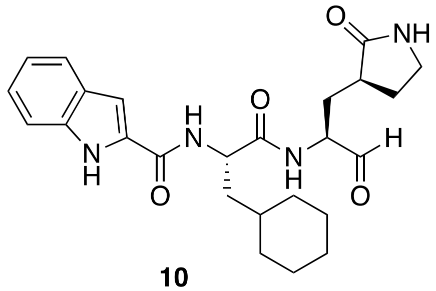

10

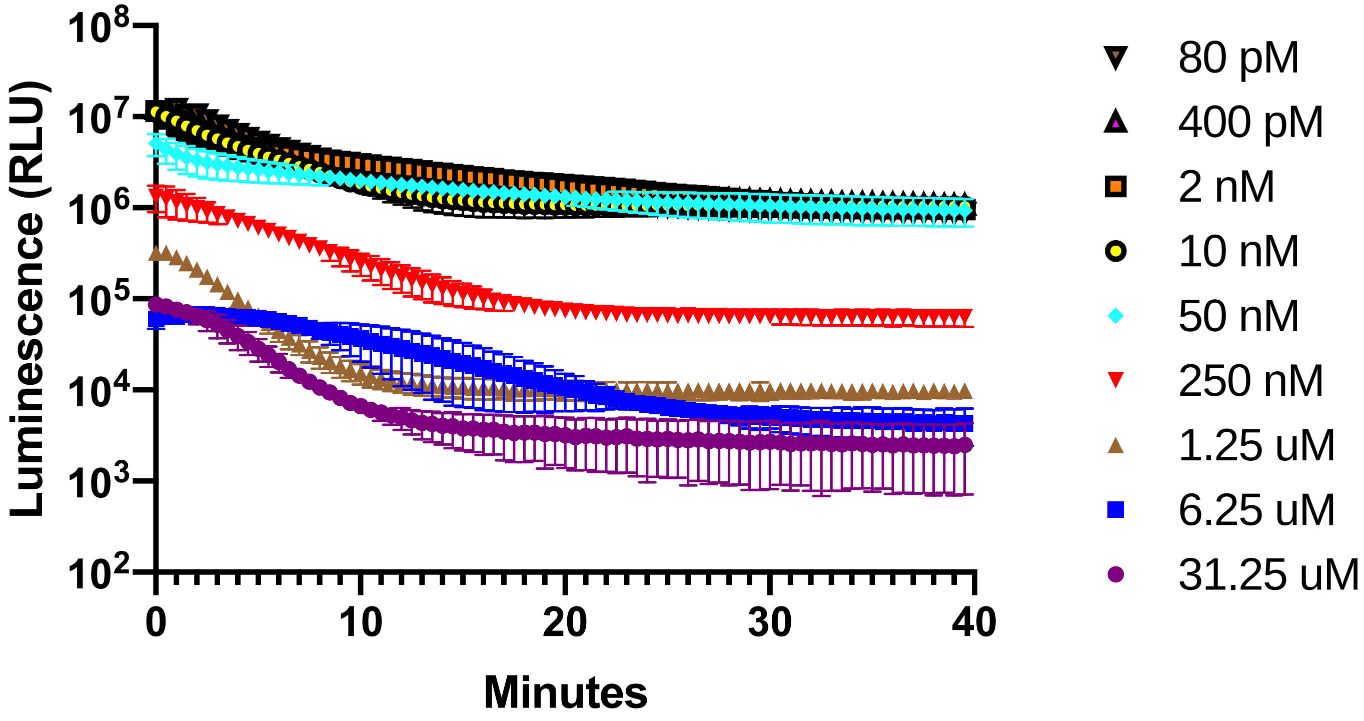

Figure S10: *Raw data of the inhibition experiments for the IC_{50} assay of compound* ***10.***

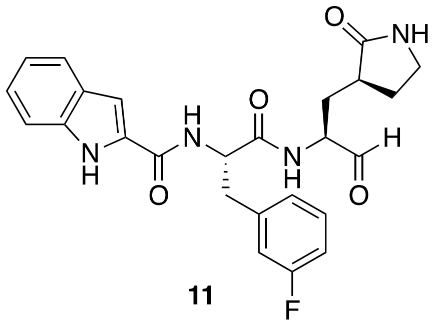

11

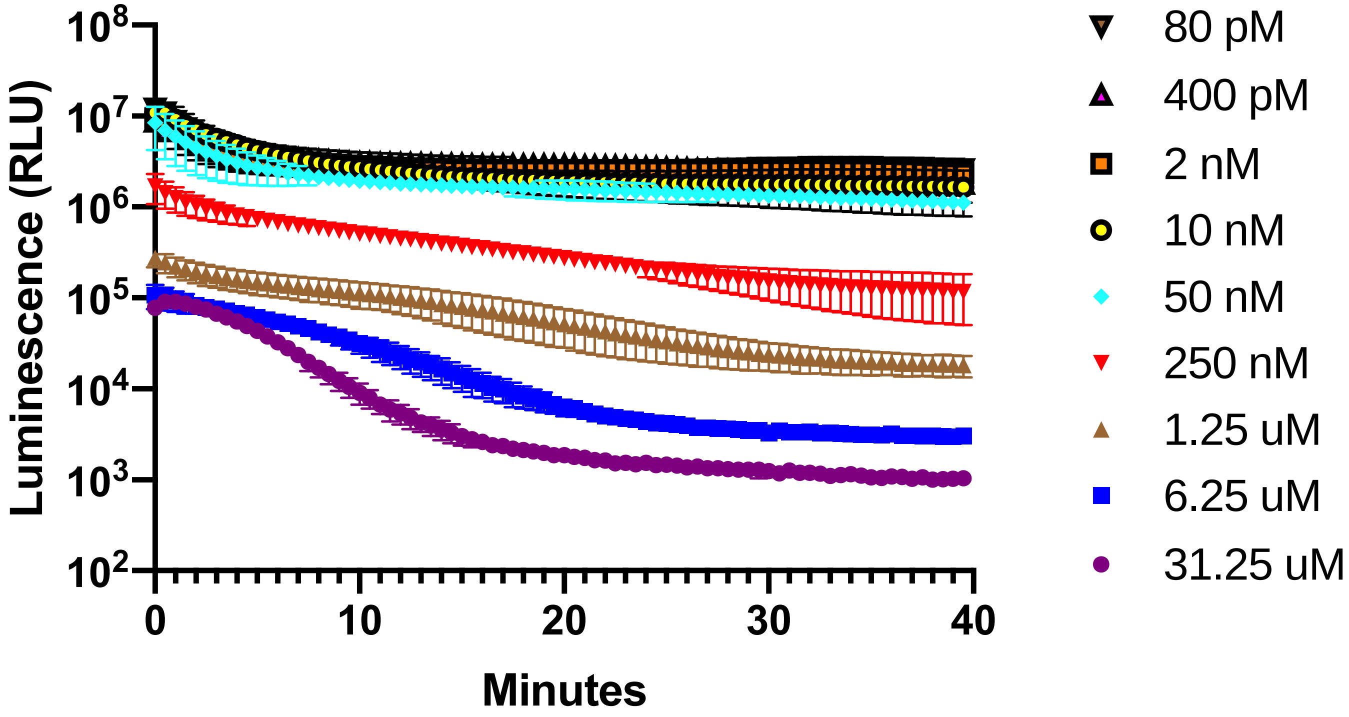

Figure S11:** Raw data of the inhibition experiments for the IC_{50} assay of compound **11.

8. Linear titration data

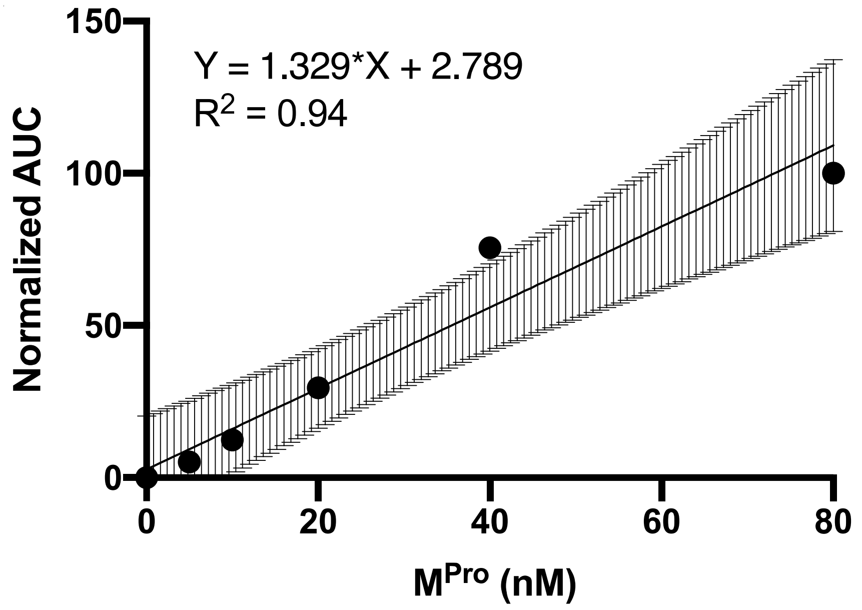

Figure S12: *Linear range of our assay (0 – 80 nM) and the corresponding regression.*

9. References

[1] J. R. Hauser, H. A. Beard, M. E. Bayana, K. E. Jolley, S. L. Warriner, R. S. Bon, *Beilstein Journal of Organic Chemistry* **2016**, *12*, 2019-2025.

[2] A. Dragulescu-Andrasi, G. Liang, J. Rao, *Bioconjugate Chem.* **2009**, *20*, 1660-1666.
